# Supplementary material for: Attitudes to Three Weight Maintenance Strategies: A Qualitative Study
Source: Nutrients. 2022 Oct 22;14(21):4441. doi: 10.3390/nu14214441 (PMC9655049; doi:10.3390/nu14214441)
Supplement: Supplementary file 1 [file nutrients-14-04441-s001.zip › nutrients-1933941-supplementary.pdf]

Supplementary material.

Reporting checklist for qualitative study.

Based on the SRQR guidelines (O'Brien BC, Harris IB, Beckman TJ, Reed DA, Cook DA. Standards for reporting qualitative research: a synthesis of recommendations. Acad Med. 2014;89(9):1245-1251.)

		Reporting item	Page number
	#1	Concise description of the nature and topic of the study identifying the study as qualitative or indicating the approach (e.g. ethnography, grounded theory) or data collection methods (e.g. interview, focus group) is recommended	**4**
	#2	Summary of the key elements of the study using the abstract format of the intended publication; typically includes background, purpose, methods, results and conclusions	**1**
Problem formulation	#3	Description and signifcance of the problem / phenomenon studied: review of relevant theory and empirical work; problem statement	**1-4**
Purpose or research question	#4	Purpose of the study and specific objectives or questions	**4**
Qualitative approach and research paradigm	#5	Qualitative approach (e.g. ethnography, grounded theory, case study, phenomenolgy, narrative research) and guiding theory if appropriate; identifying the research paradigm (e.g. postpositivist, constructivist / interpretivist) is also recommended; rationale. The rationale should briefly discuss the justification for choosing that theory, approach, method or technique rather than other options available; the assumptions and limitations implicit in those choices and how those choices influence study conclusions and transferability. As appropriate the rationale for several items might be discussed together.	**4-5**
Researcher characteristics and reflexivity	#6	Researchers' characteristics that may influence the research, including personal attributes, qualifications / experience, relationship with participants, assumptions and / or presuppositions; potential or actual interaction between researchers' characteristics and the research questions, approach, methods, results and / or transferability	**14**
Context	#7	Setting / site and salient contextual factors; rationale	**4-5**
Sampling strategy	#8	How and why research participants, documents, or events were selected; criteria for deciding when no further sampling was necessary (e.g. sampling saturation); rationale	**4**
Ethical issues pertaining to human subjects	#9	Documentation of approval by an appropriate ethics review board and participant consent, or explanation for lack thereof; other confidentiality and data security issues	**4, 14**
Data collection methods	#10	Types of data collected; details of data collection procedures including (as appropriate) start and stop dates of data collection and analysis, iterative process, triangulation of sources / methods, and modification of procedures in response to evolving study findings; rationale	**4-5**
Data collection instruments and technologies	#11	Description of instruments (e.g. interview guides, questionnaires) and devices (e.g. audio recorders) used for data collection; if / how the instruments(s) changed over the course of the study	**4-5; supplement**

Units of study	#12	Number and relevant characteristics of participants, documents, or events included in the study; level of participation (could be reported in results)	**5-6**
Data processing	#13	Methods for processing data prior to and during analysis, including transcription, data entry, data management and security, verification of data integrity, data coding, and anonymisation / deidentification of excerpts	**5**
6Data analysis	#14	Process by which inferences, themes, etc. were identified and developed, including the researchers involved in data analysis; usually references a specific paradigm or approach; rationale	**5**
Techniques to enhance trustworthiness	#15	Techniques to enhance trustworthiness and credibility of data analysis (e.g. member checking, audit trail, triangulation); rationale	**5; 14**
Syntheses and interpretation	#16	Main findings (e.g. interpretations, inferences, and themes); might include development of a theory or model, or integration with prior research or theory	**7-11**
Links to empirical data	#17	Evidence (e.g. quotes, field notes, text excerpts, photographs) to substantiate analytic findings	**7-11**
Intergration with prior work, implications, transferability and contribution(s) to the field	#18	Short summary of main findings; explanation of how findings and conclusions connect to, support, elaborate on, or challenge conclusions of earlier scholarship; discussion of scope of application / generalizability; identification of unique contributions(s) to scholarship in a discipline or field	**11-13**
Limitations	#19	Trustworthiness and limitations of findings	**13-14**
Conflicts of interest	#20	Potential sources of influence of perceived influence on study conduct and conclusions; how these were managed	**14**
Funding	#21	Sources of funding and other support; role of funders in data collection, interpretation and reporting	**14**

Topic guide

Semi-structured Interview Schedule: Part 1 – Discussing Previous Experience

Questions	Prompts and probes
What has been your experience of weight loss/weight maintenance in the past year?	What motivated you to lose/maintain weight?<br>What difficulties have you experienced during this time?<br>What factors made the experience of losing/maintaining weight easier?<br>What strategies or lifestyle changes did you use to lose/maintain weight? Why did you choose these?<br>What did you think of these? Were they useful/effective?
Now moving on slightly, imagine a scenario where you have reached your goal weight and want to avoid regaining weight and instead are trying to maintain this current weight. How might you go about this?	What strategies/lifestyle changes might you make?<br>Why would you choose these strategies? How do you think they would be useful?<br>What other factors might be helpful or unhelpful during a period of weight maintenance?

Note. Actual conversations often deviated from the interview schedule due to the semi-structured nature of the interviews.

Table 2

Semi-structured Interview Schedule: Part 2 - Discussing attitudes towards weight maintenance strategies

Questions	Prompts and probes
The first strategy suggested by people is weighing yourself before breakfast every day. What do you think of this idea?	How useful do you think it would be to you in real life?<br>What do you like or dislike about it?
The reason this has been suggested is that it is a simple way to check on whether you have been eating too much or too little in relation to maintaining a healthy goal weight, and you can do something about it. What do you think of this idea now? [a]	Would you consider using it?<br>Why/why not?
Exploring this idea further – Imagine you were weighing yourself daily and found that one day your weight had gone up compared to the day before. To counter this, you decided to try and eat less that day. How would you go about doing that?	What would you change?
Overall, how likely would you be to use daily weighing?	
Another idea about weight maintenance is missing an occasional meal. What do you think of this idea?	How useful do you think it would be to you in real life?<br>What do you like or dislike about it?
Recent studies have shown that after missing a meal you don't end up fully compensating with what you eat later. What do you think of this idea now? [b]	Would you consider using it?<br>Why/why not?

Instead of missing a meal, an alternative idea is cutting out a very high calorie food. What foods would those be for you?	Is there something you eat with your meals which might be high calorie that you could cut out?
Show Images A. These two foods have the same number of calories in them. What do you think of this?	Is this surprising?
When defining high calorie foods, we tend to mean foods that are energy dense such as chocolate, biscuits, crisps, sausage rolls. For example, you might cut out having biscuits with your tea. What do you think of this idea?	How useful do you think it would be to you in real life?<br>What do you like or dislike about it?
Show Images B and C. Both food items have the same calorie content. What do you think of this?	Is this surprising?<br>Does this make you think differently about calories?
The idea behind cutting out a high calorie habit is that it will get easier over time, the more you do it. However, it may be difficult to start or do when in a state of hunger. What do you think of this idea?	Does this change your opinion at all?
Overall, how would you rank these approaches we've discussed – missing a meal, cutting out high calorie foods, replacing high calorie habit with a low-calorie alternative?	
This is the end of the interview unless there is anything else you would like to add?	

Note. Actual conversations often deviated from the interview schedule due to the semi-structured nature of the interviews.

Depending on the comments of the participant, the researcher may have elaborated on the rationale of the approach, with possible comments such as the following:

[a] Allows sensitivity to small changes compared to the fit of your clothes, allows you to do something about your change in weight immediately as it is limited how much weight you can gain day by day, quick and easy method, help understand more about energy balance (how eating affects your weight), awareness that it is not for everyone.

[b] Able to function quite normally, don't get particularly hungry, perhaps missing breakfast would be easier than another meal as when you aren't hungry you tell yourself you could easily skip lunch, however when you are in a state of hungry it is much more difficult to actually skip lunch.

The following describes the images of food that were presented

Images A: Image of Equal Calorie Content of Two Foods: 75g of Dried Pasta (Cooked) and 26.25g of Cadbury's Dairy Milk Chocolate

Images B: Image of Equal Calorie Content of Two Foods: One Medium Apple and One McVitie's Milk Chocolate Digestive Biscuit

Images C: Image of Equal Calorie Content of Two Foods: One 175g Muller Light Strawberry Yoghurt and 22.5g of Cadbury's Dairy Milk Chocolate
